# Concentration Levels, Pollution Characteristics and Potential Ecological Risk of Dust Heavy Metals in the Metropolitan Area of Beijing, China

**DOI:** 10.3390/ijerph14101159

**Published:** 2017-09-30

**Authors:** Qiulin Xiong, Wenji Zhao, Jiayin Zhao, Wenhui Zhao, Lei Jiang

**Affiliations:** 1 Urban Environmental Process and Digital Modeling Laboratory, Capital Normal University, Beijing 100048, China; 2 College of Environmental Sciences and Enginnering, Peking University, Beijing 100871, China; jiayin.zhao@pku.edu.cn; 3 Beijing Municipal Environmental Monitoring Center, Beijing 100044, China; wenhuidiandian@163.com; 4 Beijing Municipal Research Institute of Environmental Protection, Beijing 100037, China; jiangle3657@sina.com

**Keywords:** dust heavy metals, pollution characteristics, enrichment factor, geo-accumulation index, potential ecological risk, urban-suburban areas

## Abstract

This study aims to investigate the concentration levels, pollution characteristics and the associated potential ecological risks of the heavy metals found in dust in the metropolitan area of Beijing, China during the winter. Dust samples were collected at 49 different spatial locations of Beijing’s metropolitan area from November 2013 to January 2014, in which the concentration levels of Cd, Cr, Pb, Cu, Zn, Ni, Co, V, Bi and Mo were measured by Elan DRC II type inductively coupled plasma mass spectrometry (ICP-MS). Test results showed that the concentrations of dust heavy metals Pb, Cr, Cu and Zn in the urban areas (147.1 mg·kg^−1^, 195.9 mg·kg^−1^, 239.2 mg·kg^−1^ and 713.2 mg·kg^−1^) were significantly higher than those in the suburbs (91.6 mg·kg^−1^, 125.1 mg·kg^−1^, 131.9 mg·kg^−1^ and 514.5 mg·kg^−1^). Enrichment factors and the geo-accumulation index were used to describe the pollution characteristics of dust heavy metals in urban and suburban areas. Results indicated that Zn and Cu were moderately polluting in both urban and suburban areas, Cd was severely polluting in urban areas and heavily polluting in the suburbs. Furthermore, potential ecological risk assessment revealed that the degrees of ecological harm of dust heavy metals were very strong in both urban and suburban areas, but especially in urban areas. The potential ecological risk of heavy metal Cd, whose single factor of ecological damage was extremely strong, accounted for about 90% of the total ecological risk.

## 1. Introduction

Dust refers to particulate matter in the air with an aerodynamic equivalent diameter greater than 10 microns, naturally settling to the ground due to gravity [1]. It reflects the natural subsidence amount of particles. Thus, it is generally accepted as an important environmental indicator [2]. It is the carrier of contaminants as well as containing harmful substances itself. As heavy metals are able to loosely attach to the surface of dust particles, dust presents instability and potential toxicity to some extent [3]. According to the Oxford Dictionary of Chemistry [4], “heavy metals include…copper, lead and zinc. These metals are a cause of environmental pollution from a number of sources including lead in petrol, industrial effluents, and leaching of metal ions from the soil into lakes and rivers by acid rain”. Heavy metals—especially toxic heavy metals in dust—easily settle on plants, soil and water, causing serious harm to the ecological environment and human health through the transfer and accumulation of the food chain [5,6,7]. Based on the bio-available fractions, the carcinogenic risks are mainly from the ingestion of Lead in dust. With regards to non-carcinogenic risk, accumulative multi-elements via inhalation and/or ingestion exposure can impact both children and adults, while single element ingestion such as Arsenic, Lead and Cobalt may pose risks to children [8].

In recent years, with the advance of urbanization and industrialization, the amount of urban dust–including metal pollutants—is increasing substantially [9] and seriously affecting urban air quality and the health of residents. Heavy metals (e.g., lead, mercury) are one of four categories of air pollutants and most of them are dangerous to human tissues and organs (e.g., respiratory system, cardiovascular system, nervous system, urinary system). What makes it worse is that they tend to bio-accumulate in the human body. Various studies have reported and evaluated heavy metal pollution characteristics in dust [10,11,12,13,14], analysis of pollution sources [15,16,17] and human health risks [18,19,20,21]. Research methods include cluster analysis (CA) [17], principal component analysis (PCA) [10,15,17,22], the accumulated pollution index method [21], enrichment factor (EF) analysis [15,17], Pb isotope tracer method [1,6], and health risk assessment ([18,21]). Normally, the geo-accumulation index (*I_geo_*) of heavy metals in dust is a good indicator of environmental pollution [23]. All these methods are widely used in the analysis and evaluation of atmospheric dust heavy metal pollution and its health risks.

However, the related research on the ecological environmental risk of dust heavy metals is relatively lacking [23]. Taking into account their potential threat to the environment and human health, this paper analyses winter dust heavy metals pollution conditions in Beijing using enrichment factor and geo-accumulation index methods. Then the ecological risk of dust heavy metal pollution was evaluated using the potential ecological risk index. The research results can provide a scientific basis for the control of air heavy metals pollution in urban dust.

## 2. Experimental Section

### 2.1. Dust Samples Collection

**Figure 1:**
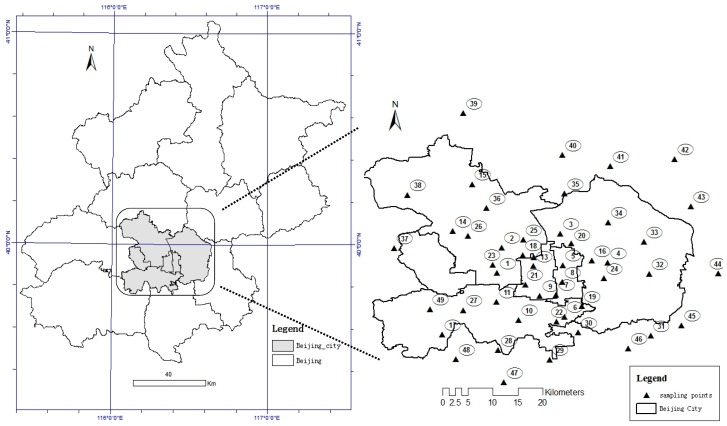
Geographic location and dust sampling points in the metropolitan area of Beijing, China.

As the capital of China, Beijing is the most famous metropolitan area in North China and has a special terrain, being surrounded on three sides by mountains from northwest to southeast. Because of its special geographical position, the climate in Beijing is a typical temperate continental monsoon climate with temperature inversion happening frequently. Atmospheric dust sampling was performed in strict accordance with the Chinese national standard [24]. A bottom-flat cylindrical tank made of glass, 30 cm high, with a 15 cm inner diameter was selected as the dust collecting cylinder. Forty-nine groups of dust samples in total were collected from sampling points distributed in Beijing city and surrounding areas, with thirteen sampling points arranged in the surrounding area as a control experiment (Figure 1). There were no tall buildings around the sampling points, and point & line local pollution sources were far away, such as main roads and chimneys. Samples were collected with clean glass cylindrical tanks at an average height of 2.5 m from the surface of the earth, and the sampling time was from 15 November 2013 to 15 January 2014, collecting two parallel samples in each site during the entire period.

Before being placed on the sample point, a small amount of ethylene glycol was added into each dry deposition cylinder (to prevent being frozen in winter, and to keep the bottom of the cylinder moist to inhibit the growth of bacteria and algae), and used to fill the bottom of the cylinder. The atmospheric dry deposition sample weight of each dry deposition collecting cylinder was between 100 mg to 1000 mg. After being collected and sealed with closures, dust samples were sent to the Institute of Oceanology of Chinese Academy of Sciences for elemental content analysis.

### 2.2. Element Content Test

Before analysis, all the atmospheric dust samples were fully grinded in agate mortar, and selected through a 100 mesh sieve. 40 mg of sample powder was placed in a PTFE bladder, which contained one 10 mg·L^−1^ multi-element standard solution mix provided by the American Company Perkin Elmer (Waltham, MA, USA). Then 0.6 mL HNO_3_, 2 mL HF was added to the agate mortar after being left to stand, and heated in an anti-corrosion oven at 150 °C for 24 h. Once cooled, 0.5 mL HClO_4_ was added, exposed and placed in the 120 °C anti-corrosion electric heating plate to dry. Next, 1 mL HNO_3_ and 1 mL H_2_O were added and sealed in an anticorrosion oven at 150 °C to dissolve back over 12 h. Finally, the solution was transferred to a polyester bottle after being cooled, and the amount of the solution was increased to 40g using ultrapure water. The trace element analysis was conducted by the professionals at the Qingdao institute of oceanography, Chinese academy of sciences, which is an authoritative element analysis and test institute in China.

The instrument used for the element test in this study was the Elan DRC II type Inductively Coupled Plasma-Mass Spectrometry (ICP-MS, Agilent, Santa Clara, CA, USA). ICP-MS is in line with the national soil pollution survey sample analysis test technical regulations and the latest requirements for soil pollution analysis test methods as prescribed by soil environment quality standard [25]. The national Marine sediment level standard substances (GBW07315, GBW07316) and USGS basalt standard substance (BCR-2, BHVO-2) were used for quality assurance of the ICP-MS analysis. The recovery percentage of standard substances wasno less than 96%. The detection limits of the analysed elements were 0.009–0.165 ng·L^−1^ (Cd 0.009 ng·L^−1^, Cr 0.125 ng·L^−1^, Pb 0.024 ng·L^−1^, Cu 0.079 ng·L^−1^, Zn 0.153 ng·L^−1^, Ni 0.103 ng·L^−1^, Co 0.078 ng·L^−1^, V 0.107 ng·L^−1^, Bi 0.023 ng·L^−1^, Mo 0.038 ng·L^−1^). The atmospheric dust elements content (mass percentage) test results covered tenheavy metals (Cd, Cr, Pb, Cu, Zn, Ni, Co, V, Bi, Mo) and one lanthanoid Tb. The data analysis of the present study was conducted using Microsoft Excel and SPSS 17.0 software (SPSS Inc., Chicago, IL, USA).

## 3. Results and Discussion

### 3.1. Concentration Levels of Heavy Metals in Atmospheric Dust of Beijing

According to the ICP-MS test results of the heavy metals in atmospheric dust samples, the statistics (minimum, maximum, extremum ratio, mean, median, standard deviation and coefficient of variation) of 10 heavy metals in winter atmospheric dust in Beijing city and the suburb area are shown in Table 1 (Beijing city) and Table 2 (Beijing suburb area). As can be seen from the tables, the content of different heavy metals in winter atmospheric dust in Beijing varied greatly. The mean content values of Cd, Bi and Mo are less than 10 mg·kg^−1^. Those of Co, Ni and V are between 10 and 100 mg·kg^−1^, accounting for a considerable portion of the atmospheric dust. The mean content values of Pb, Cr, Cu, and Zn are more than 100 mg·kg^−1^, which are the main heavy metals in the winter atmospheric dust in Beijing. The content of those heavy metals in urban areas (Pb 147.1 mg·kg^−1^, Cr 195.9 mg·kg^−1^, Cu 239.2 mg·kg^−1^, and Zn 713.2 mg·kg^−1^) is significantly higher than those in the surrounding area (Pb 91.6 mg·kg^−1^, Cr 125.1 mg·kg^−1^, Cu 131.9 mg·kg^−1^ and Zn 514.5 mg·kg^−1^), exceeding 61%, 61%, 57% and 61% respectively. This may be caused by the more dense population, and more traffic production and living activities in Beijing city compared to the surrounding areas, which shows that the contribution of human activities on dry dust heavy metals is great. In addition, the content of dust heavy metals Cr and Zn were greater than those of Lee PK’s study on Asian dust in Daejeon [6]. All four heavy metals concentrations were two times more than those in street sediments in Beijing [26].

By calculating the extreme ratio (the ratio of the maximum and the minimum values), it can be found that the concentrations distribution difference of heavy metals in Beijing winter atmospheric dust can be very large. The extreme ratio values of five heavy metals V, Ni, Co, Zn and Cu in the dust of Beijing city were between 2.3 and 6.9. Those of the other heavy metals Pb, Bi, Mo, Cr and Cd were between 12.4 and 14.6, the spatial distribution of which were definitely uneven. Compared with the extreme ratio values in Beijing city, those of heavy metals in Beijing’s surrounding area were much smaller. The extreme ratios of six heavy metals—V, Cr, Pb, Cu, Ni and Co—were between 1.4 and 2.7; those of the rest of the heavy metals—Cd, Mo, Zn and Bi—were between 3.1 and 5.3. This illustrates that the volatility of heavy metals in the urban area is more severe than in the surrounding area [10]. 

Through the above analysis, the concentration distributions of most heavy metals in winter dust in Beijing are discrete. To study the variation of these heavy metals in depth, variation coefficients (C.V) of heavy metals were calculated by using the standard deviation (SD) and mean (MN) values. The formula is C.V = (SD/MN) × 100%. The calculation results are shown in Table 1 and Table 2. According to the calculation results, the CVs of V, Ni, Co, Zn and Cu in urban dust were between 17% and 45%. Those of Bi, Mo, Cd and Pb were between 56% and 76%. Last but not least, the C.V of Cr was the highest, reaching 133%. The C.Vs of winter dust heavy metals in Beijing’s surrounding area were much smaller than those in Beijing city. Apart from Zn and Bi, the C.Vs of the other eight heavy metals were all smaller than 45%. This shows that the variability of heavy metals in the urban region is stronger than that of Beijing’s surrounding area, and the discrete degree of the observations was bigger.

### 3.2. Pollution Characteristics of Heavy Metals in Atmospheric Dust of Beijing

#### 3.2.1. Beijing Atmospheric Dust Heavy Metals Enrichment Degree

Enrichment factor (*EF*) is a common method used to study the enrichment degree of elements in atmospheric particles and dust. It can also judge and evaluate natural sources and man-made sources of such elements [27]. The enrichment factor calculation formula is as follows:

(1) $$EF={\left(C_{i}/C_{n}\right)}_{sample}/{\left(C_{i}/C_{n}\right)}_{background}$$

In Formula (1), $C_{i}$ is on behalf of the target element *i’s* concentration; $C_{n}$ is the concentration of the selected reference element *n*; ${\left(C_{i}/C_{n}\right)}_{sample}$ and ${\left(C_{i}/C_{n}\right)}_{background}$ are ratios between research element concentration and reference element concentration respectively in environmental samples and soil background [28]. With a small variation coefficient, Tb was chosen as the reference element in this study, whose content distribution in the atmospheric dust of Beijing is relatively stable [29,30]. The background value of each element is derived from the average concentration of the corresponding element in the soil of Beijing. Usually, the EF value of one element can not only reflect the level of the element’s accumulation in atmospheric dust, but also qualitatively judge and evaluate the preliminary sources of elements in atmospheric dust and their possible contribution to pollution. According to the EF values of elements in atmospheric dust, the enrichment levels of heavy metals in atmospheric dust were divided into five grades. The specific classification is shown in Table 3.

Based on the definition of the enrichment factor (EF), average EF values of ten heavy metals in the winter atmospheric dust in Beijing city and suburban areas were calculated. The calculation results are shown in Table 4. Table 4 shows that the EF values of heavy metals Bi, Cu, Ni, Pb and Cd in dust in Beijing’s urban area were almost equal to those in Beijing’s suburbs. However, the EF values of heavy metals Cr, Mo, Zn, V and Co in dust in Beijing city were higher than those in the suburbs of Beijing, which shows that heavy metals Cr, Mo, Zn, V and Co in the winter dust of Beijing city were derived more from human sources than those in the suburbs of Beijing.

The enrichment degrees of the same heavy metals in Beijing city and suburban dust were nearly the same. Four heavy metals Bi, Cu, Ni and Pb were rarely enriched, mainly coming from soil and crust sources. Another four heavy metals—Cd, Cr, Mo and Zn—were mildly enriched, coming from both natural and artificial sources. Heavy metal V was mildly enriched in the urban areas and came from both natural and artificial sources, while V was rarely enriched in the suburban areas and mainly came from soil and crust sources. Heavy metal Co was moderately enriched in urban areas and mainly came from artificial sources, while it was mildly enriched in suburban areas and came from both natural and artificial sources.

#### 3.2.2. Beijing Atmospheric Dust Heavy Metals Geo-Accumulation Index

The geo-accumulation index (*I_geo_*) is a quantitative indicator put forward by German scientist Muller in 1969 and is used to study the pollution level of heavy metals in sediments. It comprehensively considers the change of the background value caused by geological processes and the impact of human activities on the natural environment. Thus, *I_geo_* is an important index, which can both reflect the natural change characteristics of heavy metals distribution and identify the impact of human activities on the environment. In recent years, *I_geo_* is widely applied in heavy metals pollution characteristic research of soil-wind induced dust, atmospheric particulate matter and the sedimentary dust in coal-fired power plants surroundings [31]. *I_geo_* method was used in this article to analyze the pollution characteristics of ten heavy metals in the dust. The calculation formula is as follows:

(2) $$I_{geo}=\log_{2}(\frac{Cn}{1.5Bn})$$

In Formula (2), *C_n_* represents heavy metal *n’s* concentrations in dust; *B_n_* is the geochemical background value of heavy metal *n*, which is derived from the average concentration of the corresponding element in China soil [28]; 1.5 is the correction coefficient that considers the difference of background value, which is supposed to be caused by the effects of rock forming. According to the calculated *I_geo_* values, the pollution levels of heavy metals in the dust can be diagnosed. The relationship between the geo-accumulation index (*I_geo_*) and contamination rank is shown in Table 5.

According to the definition of the geo-accumulation index (*I_geo_*), average *I_geo_* values of ten heavy metals in winter atmospheric dust in Beijing city and suburban areas were calculated. The calculation results are shown in Table 6. Table 6 shows that the *I_geo_* values of most heavy metals in urban areas were greater than those of the corresponding heavy metals in the suburbs, except Cr and Co. Pollution levels and degrees of atmospheric dust heavy metals V, Co, Ni, Cr, Pb, Zn and Cu in Beijing city and suburban areas were almost the same. Whether in Beijing city or the suburban areas, the *I_geo_* values of atmospheric dust heavy metals V and Co were both smaller than 0, and the pollution levels were both non-existent. The *I_geo_* values of heavy metals Ni and Cr were both between 0 and 1, and the pollution levels were both mild pollution. The *I_geo_* value of heavy metal Pb was between 1 and 2, and the pollution level was light pollution. The *I_geo_* values of heavy metals Zn and Cu were both between 2 and 3, and the pollution levels were both moderate pollution. 

However, pollution levels and degrees of atmospheric dust heavy metals Mo, Bi and Cd in Beijing city and suburban areas were more or less different. The *I_geo_* value of heavy metal Mo in Beijing city was between 1 and 2, and the pollution level was light pollution; while that in suburban areas was between 0 and 1, and the pollution level was mild pollution. The *I_geo_* value of heavy metal Bi in Beijing city was between 2 and 3, and the pollution level was moderate pollution; while that in suburban areas was between 1 and 2, and the pollution level was light pollution. The *I_geo_* value of heavy metal Cd in Beijing city was as high as 4.2, and the pollution level was heavy pollution; while that in suburban areas was as high as 3.6, and the pollution level was high pollution. In a word, most heavy metals in Beijing urban atmospheric dust were significantly more affected by human activities compared to those in the suburbs.

### 3.3. Potential Ecological Risk Assessment of Dust Heavy Metals in Beijing

The potential ecological risk index was one quantitative index put forward by Swedish scientist Hakanson in 1980, based on the response to element abundance and the synergistic effect of pollutants [32]. It is one of the most commonly used methods for pollution level and potential ecological risk assessment of heavy metals in atmospheric particulates, soil and sediments [31]. This method not only reflects the potential ecological harm from heavy metals in single specific sediment, but also considers the integrated ecological effect of a variety of heavy metals. What is more, the method can quantitatively differentiate the potential ecological risk of heavy metals by the calculated index values. It is one comprehensive index that can represent the influence degree of heavy metals on the ecological environment. The calculation formula is as follows:

(3) $$C_{f}^{i}=\frac{C^{i}}{C_{n}^{i}},E_{r}^{i}=T_{r}^{i}\times C_{f}^{i},RI=\sum_{i}^{m}E_{r}^{i}$$

In Formula (3), $C_{f}^{i}$ is the pollution coefficient of heavy metal*i*; $C^{i}$ is heavy metal *i’s* measured concentration in the sample, whose unit is mg/kg; $C_{n}^{i}$ is heavy metal *i’s* background value [28], whose unit is mg/kg; $E_{r}^{i}$ is heavy metal *i’s* potential ecological risk coefficient; $T_{r}^{i}$ is heavy metal *i’s* toxic coefficient; RI is the total potential ecological risk index of a variety of heavy metals. An improvement has been made about the popular classification criteria of the potential ecological risk index put forward by USEPA [33], which adds “No damage”/“No risk” grade to take low values into account. The new relationship between $E_{r}^{i}$, RI and potential ecological damage is shown in Table 7.

Atmospheric dust contains a lot of harmful elements, especially toxic and persistent toxic heavy metals, which do great harm to the human body. The potential ecological risk of eight toxic heavy metals—V, Cr, Co, Ni, Cu, Zn, Cd and Pb—in the winter atmospheric dust of Beijing’s urban area and suburbs was evaluated using the potential ecological risk index method. Soil background values of V, Cr, Co, Ni, Cu Zn, Cd and Pb are 79.2, 68.1, 15.6, 29.0, 23.6, 102.6, 0.074 and 25.4 mg/kg, respectively [28]. The toxicity coefficients (TC) of V, Cr, Co, Ni, Cu, Zn, Cd and Pb are respectively 2, 2, 5, 5, 5, 1, 30, and 5 [34]. The calculation results of heavy metals potential ecological harm coefficients ($E_{r}^{i}$ and ecological risk indexes (*RI*) in the winter dust of Beijing are shown in Table 8.

Table 8 shows that the sorting of single factor potential ecological harm coefficients of winter dust heavy metals in Beijing city and suburban areas are consistent, which is V < Co < Cr < Zn < Ni < Pb < Cu < Cd. The total ecological risk index (*RI*) of eight heavy metals in winter dust of Beijing’s urban and suburban area were as high as 1104 and 678, respectively. Their corresponding ecological damages were all serious risk level, and that in Beijing city almost reaches the highest hazard rating (Extreme risk). The single factor potential ecological harm coefficient of Cd was the highest among eight heavy metals, which accounted for 89.5% and 88.3% of the total ecological risk index in urban and suburban areas, and their ecological damage both reached extreme damage level. The single factor potential ecological harm coefficients of Cu and Pb were the second highest, the sum of which respectively accounted for 7.8% and 7.5% of the total ecological risk index in urban and suburban areas. The ecological harm of Cu in urban dust reached the moderate damage level. The single factor potential ecological harm coefficient of the rest of the five heavy metals was small, the sum of which accounted for less than 5% of the total ecological risk index no matter in urban or suburban areas. The pollution coefficients and single potential ecological risks ($E_{r}^{i}$) of heavy metals Cd, Cu and Pb in the winter atmospheric dust of Beijing’s urban area were significantly higher than those corresponding values in suburban areas. The single potential ecological risks ($E_{r}^{i}$) of heavy metals Ni and Zn in winter atmospheric dust of Beijing’s urban area were slightly higher than those corresponding values in suburban areas. While the single potential ecological risks ($E_{r}^{i}$) of heavy metals Co, Cr and V in winter atmospheric dust of Beijing’s urban area were almost equivalent with those corresponding values in suburban areas.

However, in this research and other previous publications on dust heavy metals, speciation has not been considered [12,17]. This poses a problem, e.g., for chromium there are significant differences between the toxicology of tri- and hexavalent forms. Hence, their values will only give the total amount of all elements, including compounds. As the different species have large differences in toxicity, the latter cannot be reliably evaluated based on the total element content alone. In future, researchers need to combine ICP-MS with heavy metals speciation methods and take their different toxicology into account.

## 4. Conclusions

Conclusions can be drawn as follows, based on the above analysis:

(1) The content of dust heavy metals Pb, Cr, Cu and Zn in the urban areas (147.1 mg·kg^−1^, 195.9 mg·kg^−1^, 239.2 mg·kg^−1^ and 713.2 mg·kg^−1^), were significantly higher than those in the suburbs (91.6 mg·kg^−1^, 125.1 mg·kg^−1^, 131.9 mg·kg^−1^ and 514.5 mg·kg^−1^).

(2) The enrichment degrees of the same heavy metals in Beijing city and suburban dust were nearly the same. Bi, Cu, Ni and Pb, with slight enrichment, were mainly derived from the earth’s crust or soil source; while Cd, Cr, Mo and Zn, with mild enrichment, were caused by a combination of natural and artificial sources.

(3) The *I_geo_* values of most heavy metals in urban area were greater than those of the corresponding heavy metals in the suburbs except Cr and Co. But pollution levels and degrees of atmospheric dust heavy metals V, Co, Ni, Cr, Pb, Zn and Cu in Beijing city and suburban area were almost the same. The pollution levels of heavy metals Ni and Cr were both mild pollution. The pollution level of heavy metal Pb was light pollution. The pollution levels of heavy metals Zn and Cu were both moderate pollution. However, pollution levels and degrees of atmospheric dust heavy metals Mo, Bi and Cd in Beijing city and suburban area were more or less different. The pollution level of heavy metal Mo in Beijing city was light pollution; while that in suburban area was mild pollution. The pollution level of heavy metal Bi in Beijing city was moderate pollution; while that in suburban area was light pollution. The pollution level of heavy metal Cd in Beijing city was heavy pollution; while that in suburban area was high pollution. In a word, most heavy metals in Beijing urban atmospheric dust were significantly more affected by human activities compared to those in the suburbs.

(4) The total ecological damages of eight heavy metals in winter dust of Beijing urban and suburban area were all serious risk level, and that in Beijing city almost reaches the highest hazard rating (Extreme risk). The single factor potential ecological harm coefficient of Cd was the highest among eight heavy metals, which respectively accounted for nearly 90% of the total ecological risk index, and their ecological damage both reached extreme damage level. The single factor potential ecological harm coefficients of Cu and Pb were the second highest, and the ecological harm of Cu in urban dust reached the moderate damage level.

## Figures and Tables

**Table 1:** Statistics of dust Heavy metals of Beijing City in winter/mg/kg.

Heavy Metal	Min	Max	Extreme Ratio	Mean	Median	SD	C.V
Bi	0.8	9.9	13	3.4	2.8	1.9	56%
Cd	0.9	13.1	14.6	3	2.7	2.03	67%
Co	10.9	41.4	3.8	15.4	14.7	4.9	32%
Cr	85.9	1242.1	14.5	195.9	130.4	259.72	33%
Cu	75.9	523.7	6.9	239.2	218.5	108.6	45%
Mo	2.9	38.6	13.4	9.9	9.5	5.98	61%
Ni	30.7	95.8	3.1	61.2	54.4	16.97	28%
Pb	40.3	500	12.4	147.1	109.6	111.26	76%
V	53.4	124.1	2.3	80.7	80	13.58	17%
Zn	234.6	1000	4.3	713.2	654.3	249.18	35%
Tb	0.4	0.9	2.3	0.6	0.6	0.09	15%

C.V: variation coefficients.

**Table 2:** Statistics of dust Heavy metals of Beijing suburban in winter/mg/kg.

Heavy Metal	Min	Max	Extreme Ratio	Mean	Median	SD	C.V
Bi	0.9	4.6	5.3	1.9	1.7	0.97	50%
Cd	1.1	3.4	3.1	1.9	1.8	0.64	34%
Co	12.7	34.4	2.7	17.7	15.1	7.27	41%
Cr	92.9	199	2.1	125.1	109.1	35.92	29%
Cu	85.9	200.5	2.3	131.9	114.8	39.68	30%
Mo	3.3	11.6	3.5	5.3	4.6	2.24	42%
Ni	34.6	80.5	2.3	48.8	44.9	11.78	24%
Pb	61.8	136.1	2.2	91.6	86.6	22	24%
V	63.6	90	1.4	80.9	81.7	7.34	9%
Zn	262.8	1000	3.8	514.5	433.7	246.05	48%
Tb	0.5	0.7	1.6	0.6	0.6	0.07	11%

C.V: variation coefficients.

**Table 3:** Relationships of EF and enrichment degree of the chemical elements in dust.

EF Values	EF ≤ 1	1 < EF ≤ 10	10 < EF ≤ 100	100 < EF ≤ 1000	EF > 1000
Level	Rarely enriched	Mildly enriched	Moderately enriched	Highly enriched	Extremely enriched
Rank	1	2	3	4	5
Source	Soil and crust source	Natural and artificial sources	artificial source	artificial source	artificial source

EF: Enrichment factor.

**Table 4:** The enrichment factor of dust heavy metals during winter in Beijing.

Heavy Metal	Reference	Urban Dust			SuburbanDust		
		Mean	EF	Level	Mean	EF	Level
Bi	0.4	2.2	1	rarely enriched	3	1	rarely enriched
Cd	0.1	5.3	2	mildly enriched	2.7	2	mildly enriched
Co	12.7	10.5	12	moderately enriched	16	6	mildly enriched
Cr	61	91.5	10	mildly enriched	177.1	7	mildly enriched
Cu	22.6	107.1	1	rarely enriched	210.7	1	rarely enriched
Mo	2	5.6	3	mildly enriched	8.7	2	mildly enriched
Ni	26.9	35.8	1	rarely enriched	57.9	1	rarely enriched
Pb	26	177.2	1	rarely enriched	132.4	1	rarely enriched
V	75.5	80.7	2	mildly enriched	59.2	1	rarely enriched
Zn	74.2	822	6	mildly enriched	660.5	3	mildly enriched

Note: Each unit of Reference value and Mean value is mg/kg.

**Table 5:** Contamination rank corresponding to geo-accumulation index.

*I_geo_* Values	*I_geo_* ≤ 0	0 < *I_geo_* ≤ 1	1 < *I_geo_* ≤ 2	2 < *I_geo_* ≤ 3	3 < *I_geo_* ≤ 4	4 < *I_geo_* ≤ 5	*I_geo_* > 5
Rank	0	1	2	3	4	5	6
Pollution levels	No pollution	Mild pollution	Light pollution	Moderate pollution	High pollution	Heavy pollution	Extreme pollution

**Table 6:** The geo-accumulation index of dust heavy metals during winter in Beijing.

Heavy Metal	Reference	Urban Dust			Surburban Dust		
		*I_geo_*	Rank	Pollution Level	*I_geo_*	Rank	Pollution Level
Bi	0.4	2.3	3	Moderate pollution	1.7	2	Light pollution
Cd	0.1	4.2	5	Heavy pollution	3.6	4	High pollution
Co	12.7	−0.4	0	No pollution	−0.3	0	No pollution
Cr	61	0.6	1	Mild pollution	0.6	1	Mild pollution
Cu	22.6	2.7	3	Moderate pollution	2	3	Moderate pollution
Mo	2	1.6	2	Light pollution	0.8	1	Mild pollution
Ni	26.9	0.5	1	Mild pollution	0.3	1	Mild pollution
Pb	26	1.7	2	Light pollution	1.2	2	Light pollution
V	75.5	−0.5	0	No pollution	−0.9	0	No pollution
Zn	74.2	2.6	3	Moderate pollution	2.2	3	Moderate pollution

**Table 7:** Improved classification criteria of the potential ecological risk index.

$E_{r}^{i}$	Single Ecological Damage	RI	Total Ecological Risk
<10	No damage	<50	No risk
10–40	Mild damage	50–150	Mild risk
40–80	Moderate damage	150–300	Moderate risk
80–160	High damage	300–600	High risk
160–320	Serious damage	600–1200 >1200	Serious risk Extreme risk
>320	Extreme damage		

$E_{r}^{i}$: potential ecological risk coefficient; RI: total potential ecological risk index.

**Table 8:** The potential ecological risk index of heavy metals of dust during winter in Beijing.

Heavy Metal	TC	Urban Dust			Suburban Dust		
		PC	$E_{r}^{i}$	ED	PC	$E_{r}^{i}$	ED
Cd	30	33	988	Extreme damage	20	599	Extreme damage
Co	5	1	5	No damage	1	5	No damage
Cr	2	3	5	No damage	3	5	No damage
Cu	5	11	56	Moderate damage	6	29	Mild damage
Ni	5	2	11	Mild damage	2	9	No damage
Pb	5	6	30	Mild damage	4	22	Mild damage
V	2	1	2	No damage	1	2	No damage
Zn	1	7	7	No damage	6	6	No damage
*RI*	-	-	1104	Serious risk	-	678	Serious risk

TC: toxicity coefficient; PC: pollution coefficient; ED: ecological damage.
